# Cranial and Systemic Manifestations of Giant Cell Arteritis: Two Sides of the Same Coin

**DOI:** 10.7759/cureus.108168

**Published:** 2026-05-03

**Authors:** Léonard Zufferey, Chloé Manca, Fatma Ouamer, Nicolas Garin, Laura Moi

**Affiliations:** 1 Department of Internal Medicine, Hôpital Riviera-Chablais, Rennaz, CHE; 2 Department of Nuclear Medicine, Hôpital Riviera-Chablais, Rennaz, CHE; 3 Department of Neuroradiology, Hôpital Riviera-Chablais, Rennaz, CHE; 4 Division of Allergy and Immunology, Institut Central de l'Hôpital du Valais, Sion, CHE

**Keywords:** giant cell arteritis, glucocorticoid therapy, horton disease, polymyalgia rheumatica, vasculitis

## Abstract

Giant cell arteritis (GCA) is the most common form of vasculitis in people over the age of 50. It constitutes a true medical emergency due to the risk of blindness or other ischemic manifestations. Treatment should therefore ideally begin within hours of the onset of the first clinical signs suggestive of this diagnosis. However, the often complex and nonspecific clinical presentation, combined with the absence of clear diagnostic criteria, frequently leads to delays in management. We report two cases of patients with giant cell arteritis presenting with very distinct manifestations. The first case is a classic history of Horton’s disease, rapidly diagnosed due to the typical clinical presentation. In the second case, the diagnosis required additional imaging studies, notably a PET-CT scan, due to the nonspecific yet common presentation of giant cell arteritis, which should not be overlooked. Through these two cases, we aim to raise awareness among primary care clinicians regarding the various clinical presentations that should raise suspicion of giant cell arteritis and prompt the need for further investigations.

## Introduction

Giant cell arteritis (GCA) is the most common form of vasculitis in people over the age of 50. It predominantly affects women, with an estimated prevalence in Europe and North America of 1 in 500 people and an annual incidence of 20 to 30 cases per 100,000 people [1]. It is a granulomatous inflammation of the vascular wall, mediated by CD4+ lymphocytes and macrophages via cytokines such as IL-6 and interferon-γ, which activate the Janus kinase/signal transducers and activators of transcription (JAK/STAT)pathway [1]. It primarily affects large and medium-sized arteries, particularly the cranial branches of the aorta, such as the temporal arteries, and represents a significant risk of ischemia if diagnosis is delayed [2].

Clinical manifestations are highly variable, ranging from classic Horton’s disease (headaches, scalp hyperesthesia, jaw claudication, and visual disturbances) to nonspecific systemic symptoms including fever, asthenia, weight loss, and occasionally arthralgia similar to polymyalgia rheumatica. The diagnosis of GCA is therefore complex, especially since no reliable specific test is currently available. Imaging studies are thus key tools for rapid diagnosis and monitoring, allowing for prompt initiation of treatment and prevention of ischemic complications [3].

Treatment initially involves high-dose corticosteroid therapy, which should be started as soon as possible. However, due to frequent relapses following tapering of corticosteroids and the side effects of such long-term treatment, it is necessary to introduce corticosteroid-sparing biologics such as tocilizumab (anti-IL-6) as soon as possible [3-19].

In this article, we report two cases of giant cell arteritis with very distinct classic presentations, one of the cephalic form and the other of the systemic form, in order to raise awareness among primary care clinicians regarding the various classic manifestations of this common disease and to highlight the diagnostic challenge that this condition can pose.

## Case presentation

Case 1

An 84-year-old female patient, with a history of atrial fibrillation and Crohn's disease, was admitted to the emergency department with headaches and visual disturbances. She reported right peri-orbital headaches that had been progressively worsening for three weeks, accompanied for the past two weeks by the appearance of an opaque veil affecting the right upper visual field when using monocular vision. There was no jaw claudication, trismus, scalp tenderness, or general symptoms. An ophthalmological assessment carried out two weeks earlier on an outpatient basis revealed right anterior ischaemic optic neuropathy, with preserved visual acuity.

Neurological examination was unremarkable. There were no cranial nerve deficits or objective visual impairment. Laboratory tests showed an erythrocyte sedimentation rate (ESR) of 87 mm/h and C-reactive protein (CRP) of 12.2 mg/L (Table 1). The lumbar puncture, performed to rule out encephalitis, reveals moderate proteinorachia, with no other abnormalities, and anti-neuronal and anti-myelin oligodendrocyte glycoprotein (anti-MOG)antibodies were negative. There was no halo sign on the ultrasound of the temporal arteries. Brain CT angiography revealed grade I right exophthalmos and right retro-ocular optic perineuritis. Cerebral MRI showed multifocal arteritis affecting the ophthalmic and temporal territories, bilateral but predominantly on the right side (Figures 1, 2, 3). The temporal artery biopsy, performed secondarily, was negative, probably due to suboptimal sample length.

**Table 1:** Vital signs and laboratory findings of Case 1 ESR: erythrocyte sedimentation rate; CRP: C-reactive protein

		Case 1 values	Normal values
Vital signs	Blood pressure	144/62 mmHg	110-140/70-90 mmHg
	Heart rate	57 bpm	60 – 100 bpm
	Temperature	36.2°C	35.5 – 38°C
Laboratory findings	ESR	87 mm/h	1 – 20 mm/h
	CRP	12.2 mg/L	< 5 mg/L

**Figure 1:**
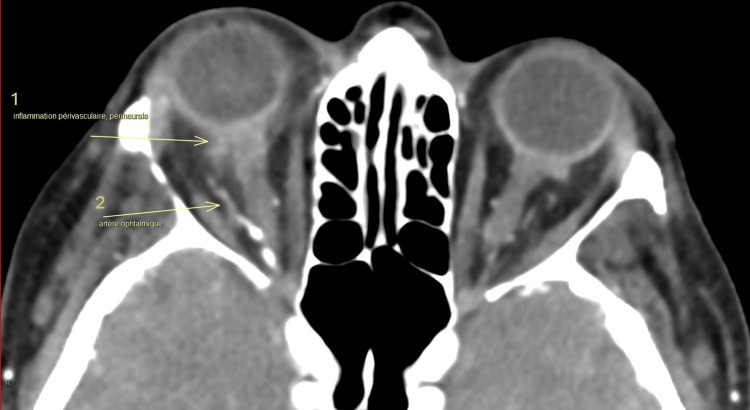
CT angiography shows right retro-ocular optic perineuritis (inflammatory infiltration of the perivascular fat) and right ophthalmic artery inflammation (blurred contours of the arterial wall), consistent with ocular involvement in the ocular form of GCA 1: perivascular and perineural inflammation; 2: ophthalmic artery GCA: giant cell arteritis

**Figure 2:**
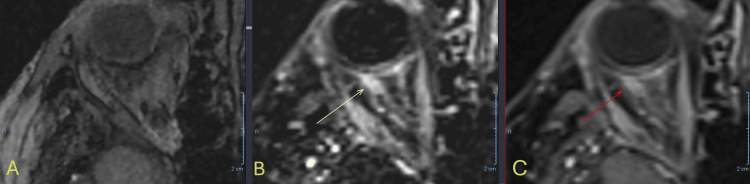
Right retro-ocular optic perineuritis on the cerebral MRI, with parietal arterial thickening and wall enhancement following gadolinium injection on T1-weighted images A: T2 sequence; B and C: T1 sequence before and after gadolinium injection, respectively.

**Figure 3:**
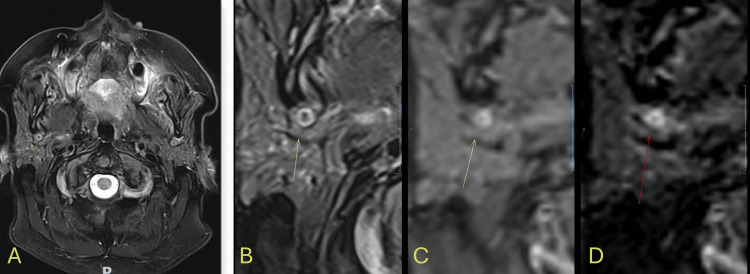
Right temporal artery inflammation on the cerebral MRI A and B: T2 sequence; C and D: T1 sequence before and after gadolinium injection, respectively. The arrows point to the right temporal artery.

In this context, following a pre-immunosuppression screening (for tuberculosis, HIV, hepatitis B virus (HBV), and hepatitis C virus (HCV)), oral high-dose glucocorticoid therapy (prednisone 60 mg/day) was initiated, resulting in rapid improvement, with regression of headaches and visual symptoms within a week and stabilization of ophthalmological findings. Despite the patient not receiving induction therapy with methylprednisolone pulses, the course remains favorable. One month later, the patient reported no visual or neurological symptoms while tapering the prednisone regimen. The ophthalmology examination showed papillary pallor with resolution of oedema, peripapillary atrophy, as well as remodeling of the superior macular pigment epithelium. The visual field examination revealed persistence of a coeco-central scotoma, for which the patient was asymptomatic in binocular vision. Tocilizumab (162 mg weekly) was started 5 weeks after diagnosis.

Case 2

A 66-year-old man with no significant past medical history was admitted to the emergency department with a two-week history of general health deterioration. He reported moderate holocranial headaches, incompletely relieved by paracetamol, associated with diffuse, symmetric inflammatory polyarthralgia predominantly involving the shoulders and hips, and a weight loss of 4 kg in 3 weeks. There was no associated fever, chills, night sweats, or other infectious symptoms.

On admission, the vital parameters and physical examination were unremarkable. In particular, there was no sign of a focal infectious or inflammatory disease. Laboratory tests revealed a CRP level of 90 mg/L and an ESR of 68 mm/h, and mild elevation of liver function tests (Table 2). The cerebrospinal fluid analysis was normal, ruling out an autoimmune disease of the central nervous system. An extensive screening for autoimmune and infectious diseases (including anti-neutrophil cytoplasmic antibodies (ANCA), anti-cyclic citrullinated peptide antibodies (anti-CCP), antinuclear and antinucleosome antibodies, as well as serological tests for *Borrelia*, *Coxiella burnetii*, *Treponema pallidum*, Epstein-Barr virus (EBV), HIV, cytomegalovirus (CMV), hepatitis A virus (HAV), HBV, HCV, hepatitis E virus (HEV), *Toxoplasma*, leptospirosis, and tularemia) was negative. CT scans of the main branches of the aorta, temporal artery ultrasound, and liver ultrasound were unremarkable and reasonably ruled out solid neoplasia and a deep infectious source.

**Table 2:** Vital signs and laboratory findings of Case 2 ALAT: alanine aminotransferase; ASAT: aspartate aminotransferase; bpm: beats per minute; CRP: C-reactive protein; ESR: erythrocyte sedimentation rate; Gamma-GT: Gamma-glutamyl transferase; ALP: alkaline phosphatase

		Case 2 values	Normal values
Vital signs	Blood pressure	134/83 mmHg	110-140/70-90 mmHg
	Heart rate	68 bpm	60 – 100 bpm
	Temperature	37.6°C	35.5 – 38°C
Laboratory findings	ESR	64 mm/h	1 – 20 mm/h
	CRP	89.9 mg/L	< 5 mg/L
	ALAT	148 U/L	< 50 U/L
	ASAT	114 U/L	< 50 U/L
	Gamma-GT	193 U/L	< 60 U/L
	ALP	184 U/L	< 130 U/L
	Total bilirubin	5.7 µmol/L	< 17 µmol/L

Due to the severity and persistence of systemic symptoms and inflammation in an elderly patient, a positron emission tomography-computed tomography (PET-CT) scan was performed 5 weeks after symptom onset and revealed significant arterial wall hypermetabolism, consistent with vasculitis affecting the aorta, the bilateral proximal subclavian arteries and axillary arteries, as well as the right common carotid artery and right vertebral arteries (Figure 4), strongly suggesting giant cell arteritis (total vascular score (TVS) 14/21).

**Figure 4:**
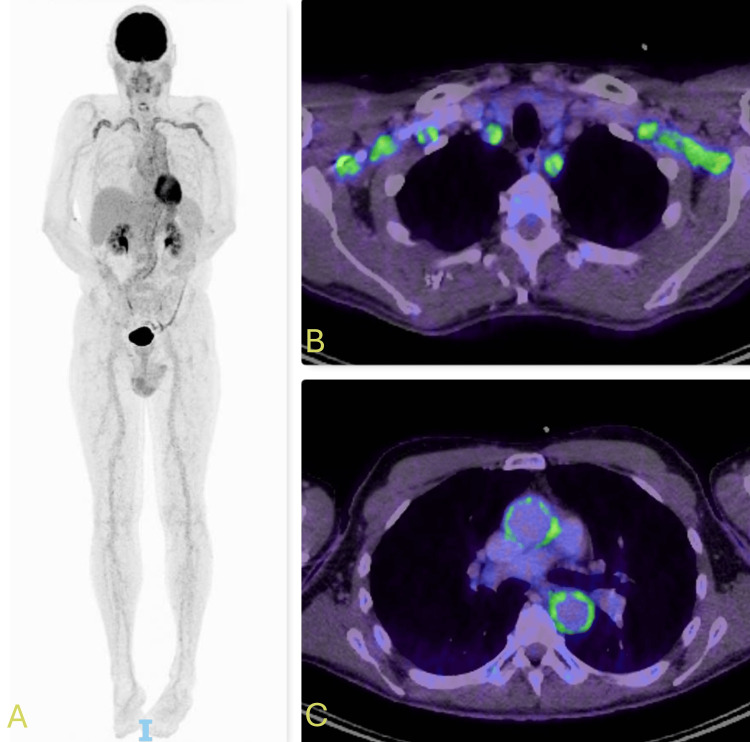
Significant hypermetabolism of the walls of the aorta, bilateral proximal subclavian and axillary arteries, and the right common carotid artery A: Scintigraphy showing significant hypermetabolism of the aortic walls, bilateral subclavian and proximal axillary arteries, and minimal hypermetabolism of the right common carotid and right vertebral arteries. B: 18F-FDG PET-CT showing significant hypermetabolism of the bilateral subclavian and proximal axillary arteries. C: 18F-FDG PET-CT showing significant hypermetabolism of the ascending and descending aorta. Total Vascular Score (TVS): 14/21 (thoracic aorta: grade 3, abdominal aorta: grade 3, subclavian arteries: grade 3, axillary arteries: grade 2, carotid arteries: grade 3, iliac arteries: grade 0, femoral arteries: grade 0). 18F-FDG PET-CT: 18F-fluorodeoxyglucose positron emission tomography - computed tomography

A diagnosis of large-vessel vasculitis was made, and, following a pre-immunosuppression screening (for tuberculosis, HIV, HBV, and HCV), oral prednisone (60 mg daily) was initiated for three weeks, followed by a gradual taper over several weeks, along with calcium-vitamin D supplementation and prophylactic trimethoprim-sulfamethoxazole. The early clinical course was favorable, and he was discharged in good clinical condition. Five months after diagnosis, the clinical and biological evolution was still favorable under prednisone in a tapering regimen and tocilizumab.

## Discussion

Giant cell arteritis (GCA) is the most common vasculitis in adults over the age of 50, mainly affecting women and Caucasians. Its global prevalence is estimated at around 20-30 cases per 100,000 people over the age of 50, with incidence varying by region, particularly higher in Northern Europe and North America [1-2].

Giant cell arteritis can present in two distinct forms [3]: the well-known cranial form (Horton's disease), which often has a sudden onset with temporal headaches, scalp hyperesthesia, temporal sensitivity, jaw claudication, and visual disturbances (which can lead to blindness); and the more insidious systemic form, which combines general symptoms (moderate fever, weight loss, asthenia) with inflammatory pain in the shoulder and pelvic girdles, consistent with polymyalgia rheumatica. Overlap between cranial and extra-cranial GCA is frequent, with up to 50% of patients suffering from cranial GCA also having constitutional symptoms. In around 15% of patients, these symptoms may dominate the clinical presentation at the time of diagnosis [4]. The coexistence of both forms is evidenced by the fact that over half of patients with biopsy-confirmed giant cell arteritis (GCA) have large-vessel involvement [5-6]. There are criteria for classifying GCA, published in 2022 by the European League Against Rheumatism (EULAR), which are useful for guiding the diagnosis of GCA but are not very sensitive, as they do not rule out GCA if the criteria are not met [7].

There are no specific clinical signs for GCA. However, temporal artery induration can be found in 30% of cases of the cephalic form [8], and blood pressure asymmetry in both arms in 15-20% of cases of the systemic form [9]. There are also no specific biological criteria for GCA, although ESR and CRP are frequently elevated in both forms (95% of cases) [10]. The two forms of GCA also differ in terms of time to diagnosis, with an average duration of 3-4 weeks for the cephalic form compared to 12-17 weeks for the systemic form [11], which may explain why vascular complications are more common in the systemic form. The main complications of the cephalic form are blindness (15-20% in cases of delayed diagnosis) and stroke (1.5-7%) [12]. Complications of the systemic form mainly include aneurysms and dissections of large arterial trunks (25-30%) [13]. The mortality rate for GCA is similar to that of the general population, except in cases of vascular complications, particularly aortic involvement in the systemic form. These differences are summarized in Table 3.

**Table 3:** Comparison of the cranial and systemic forms of GCA - half of the patients present with overlap of the two forms Data compiled by the authors from the following sources: [8, 9, 10, 11, 12, 13]. GCA: giant cell arteritis; CRP: C-reactive protein; ESR: erythrocyte sedimentation rate; FDG-PET: fluorodeoxyglucose-positron emission tomography; MRI:  magnetic resonance imaging; US: ultrasonography

	Cranial form (Horton)	Systemic form
Symptoms	Headaches, jaw claudication, scalp hyperesthesia, visual symptoms (vision loss, diplopia, hallucinations)	Fever, asthenia, weight loss, polymyalgic pain, claudication of the lower limbs
Clinical signs	Temporal artery tenderness (30%) [8]	Blood pressure asymmetry in both arms (15-20%) [9]
Laboratory	Elevation of ESR and CRP (95%) [10]	
Imaging tests	US of axillary and temporal arteries (halo sign), MRI angiography of cranial and extracranial vessels	Axillary artery US, FDG-PET
Pathology	Temporal artery biopsy	-
Time to diagnosis	3-4 weeks [11]	12-17 weeks [11]
Treatment	Corticosteroid therapy IV or orally in a tapering regimen, followed by tocilizumab	
Morbidity	Risk of blindness (15–20%) and stroke (1.5–7%) [12]	Risk of aneurysm or dissection of large arterial trunks (25–30%) [13]
Mortality	Similar to the general population, if there is no ischaemic complication	

Given that there are no specific clinical and biological criteria for GCA, imaging tests are often necessary to make a diagnosis. The Doppler ultrasound, recommended as the first-line imagery modality by EULAR, detects inflammation of the cranial and axillary arteries via the halo sign (>0.3 mm), with a sensitivity of 78-93% and a specificity of 91-96%. It is accessible, inexpensive, and radiation-free, but remains operator-dependent and has low sensitivity for large vessels [14]. 18F-FDG PET-CT allows for whole-body assessment, with a sensitivity of 71-92% and a specificity of 85-100% for cranial arteries, and 83.5-89% and 85.1-98% for large vessels (up to 89% and 98% combined), which is particularly useful in the systemic form, However, its cost, radiation exposure and and accessibility limit its use, especially since its sensitivity decreases after 72 hours of corticosteroid therapy. High-resolution MRI, a radiation-free alternative, shows a sensitivity of 68-89% and a specificity of 73-97% for cranial arteries (78.4% and 90.4% in a multicenter study) and 81% and 98% for large vessels, but its sensitivity also decreases after 72 hours of corticosteroid treatment.

Finally, computed tomography angiography provides excellent anatomical analysis and detects complications, with a sensitivity of 71.4-90% and a specificity of 85.7-100% for cranial arteries, and 67% and 98% for the aorta, at the cost of radiation exposure and iodinated contrast. A 2024 article concludes that ultrasonography (US)-Doppler, positron emission tomography (PET)-CT, MRI angiography, and CT angiography have similar specificities, but that US-Doppler has increased sensitivity [15]. Temporal artery biopsy remains the gold standard but is no longer mandatory according to EULAR recommendations, given that the diagnosis can be supported by alternative diagnostic imaging (US, PET-CT). Furthermore, a negative biopsy does not rule out GCA, as the inflammation is generally segmental. Pathologic findings can be found up to years after the introduction of immunosuppressive therapy; however, in 30% of patients, signs of arteritis will no longer be present after three months [16-17]. 

Despite these considerations, diagnosing GCA, and particularly the systemic form, remains challenging, largely because the clinical presentation and laboratory findings are highly nonspecific and consistent with many other conditions. It is therefore necessary first to rule out infectious diseases (including tuberculosis and systemic viral infections such as CMV, EBV, or HIV), malignant neoplasms and hematologic malignancies, as well as systemic inflammatory diseases (rheumatoid arthritis, adult Still’s disease, or systemic lupus erythematosus) before considering this diagnosis. It is primarily the severity and persistence of general symptoms associated with a marked inflammatory syndrome in an elderly patient in whom the main differential diagnoses have been ruled out that should raise suspicion of systemic giant cell arteritis and lead to the performance of a PET-CT scan to investigate for vasculitic involvement.

First-line therapy is based on intravenous (IV) or oral (PO) glucocorticoids, depending on the severity of the ophthalmological manifestations (methylprednisolone 250 to 1000 mg IV for 3 days in cases of ocular involvement, or prednisone 40-60 mg/day PO without visual impairment). This treatment provides rapid improvement of symptoms and prevents blindness. However, relapses are common when glucocorticoids are tapered off, and adverse effects of these long-term treatments are frequent [3-18]. Steroid-sparing treatments such as tocilizumab (anti-IL6 receptor) are recommended in case of relapse or corticosteroid dependence [19]. Methotrexate may be considered, but it seems less effective in maintaining remission and preventing relapse [20]. More recently, upadacitinib (JAK1 inhibitor) has shown efficacy in a phase 3 trial.

Long-term safety and efficacy for this indication are yet to be determined [1]. Before initiating these therapies, a pre-immunosuppression workup must be performed, including screening for tuberculosis and serological testing for HIV, HBV, and HCV. In cases of prolonged corticosteroid therapy, close monitoring of blood glucose levels to detect corticosteroid-induced diabetes and supplementation with calcium and vitamin D to prevent corticosteroid-induced osteoporosis must be implemented. Prophylaxis against *Pneumocystis jirovecii *may also be considered in accordance with specific protocols. It should be noted that in cases of suspected GCA with symptoms of ophthalmic ischemia, the introduction of corticosteroid therapy should not be delayed awaiting radiological examination or temporal artery biopsy.

## Conclusions

Giant cell arteritis (GCA) remains the most common vasculitis in patients over 50 years of age, but its diagnosis remains complex due to its highly variable clinical manifestations and the absence of specific clinical and biological diagnostic criteria. However, this diagnosis should be considered promptly in patients over 50 years with persistent severe systemic symptoms and inflammation, despite negative results from diagnostic testing for common causes. Additional radiological examinations, particularly PET-CT, should be considered in order to rule out or confirm a GCA and start glucocorticoid therapy as soon as possible. However, these examinations should not delay the introduction of a treatment in cases of visual impairment.
